# Cloned Myogenic Cells Can Transdifferentiate *In Vivo* into Neuron-Like Cells

**DOI:** 10.1371/journal.pone.0008814

**Published:** 2010-01-21

**Authors:** Rachel Sarig, Ora Fuchs, Lilach Tencer, Avi Panski, Uri Nudel, David Yaffe

**Affiliations:** 1 Department of Molecular Cell Biology, Weizmann Institute of Science, Rehovot, Israel; 2 Department of Orthopedics, Kaplan Hospital, Rehovot, Israel; Universidade Federal do Rio de Janeiro (UFRJ), Brazil

## Abstract

**Background:**

The question of whether intact somatic cells committed to a specific differentiation fate, can be reprogrammed *in vivo* by exposing them to a different host microenvironment is a matter of controversy. Many reports on transdifferentiation could be explained by fusion with host cells or reflect intrinsic heterogeneity of the donor cell population.

**Methodology/Principal Findings:**

We have tested the capacity of cloned populations of mouse and human muscle progenitor cells, committed to the myogenic pathway, to transdifferentiate to neurons, following their inoculation into the developing brain of newborn mice. Both cell types migrated into various brain regions, and a fraction of them gained a neuronal morphology and expressed neuronal or glial markers. Likewise, inoculated cloned human myogenic cells expressed a human specific neurofilament protein. Brain injected donor cells that expressed a YFP transgene controlled by a neuronal specific promoter, were isolated by FACS. The isolated cells had a wild-type diploid DNA content.

**Conclusions:**

These and other results indicate a genuine transdifferentiation phenomenon induced by the host brain microenvironment and not by fusion with host cells. The results may potentially be relevant to the prospect of autologous cell therapy approach for CNS diseases.

## Introduction

Recent studies showed that a variety of somatic cell types can be reprogrammed into pluripotent cells (iPS cells), closely resembling embryonic stem cells, by exposing their genome to transcription factors which activate endogenous genes involved in the maintenance of stem cells pluripotency [e.g. 1]. Yet, the question of whether genetically unmodified intact cells that are already committed to a specific differentiation default can be reprogrammed to a different fate by ectopic microenvironmental cues, is still a matter of controversy [e.g. 2]–[4]. Many reported cases of transdifferentiation following inoculation of cells into a suitable host tissue could be explained on the basis of fusion with a host cell committed to a different differentiation program, which overrides the program of the donor cell [5], [6]. In addition, data accumulated indicate the prevalence of multipotent stem cells in many adult tissues. Therefore, the apparent plasticity could, in some cases, be due to the differentiation of uncommitted cells which originated from the donor tissue.

Muscle progenitor cells (MPCs) are an easily accessible cell type with well-characterized markers associated with its various differentiation stages. It is also relatively simple to clone and manipulate them in culture; thus offering a convenient model system to study the differentiation plasticity of mammalian cells. Muscle progenitor cells are also promising candidates for the treatment of muscle degenerative diseases and perhaps also as a source for replacement of other cell types, provided that they can be reprogrammed into different fates. The mononucleated progenitors of the skeletal muscle, the myoblasts of the growing muscle, were among the first examples to demonstrate the extreme stability of a differentiation program. Clonal analysis in cell culture showed the stable retention, during many cell generations, of a committed program of self renewal and a default for myogenic terminal differentiation [7]–[9]. There have been several reports on the isolation of cells from muscle tissue that are capable of differentiation into a variety of cell types including neuron-like cells. However, in those cases the donor cells seemed to contain subpopulations of cells not committed to the myogenic lineage, that reside in skeletal muscle [10]–[15]. In view of the importance of the basic biological question, and its possible relevance to the prospect of cell therapy, we examined this question by using characterized cloned populations of myogenic cells expressing the muscle specific transcription factor MyoD, and manifesting the default to differentiate into muscle fibers and to participate in muscle regeneration [16]. Such mouse myogenic cells and cloned human myoblasts were inoculated into the developing brain of newborn mice. We have found that a significant fraction of the inoculated cells spread in the brain and transdifferentiated into neuronal-like cells expressing neuronal markers, without fusion with host cells, thus indicating a genuine transdifferentiation process induced by the host developing brain environment.

## Materials and Methods

### Ethics Statement

Research involving human participants was approved by the Helsinki Committee (H.C.) of the Israeli Ministry of Health and by the Kaplan Hospital H.C. Clinical investigations (muscle biopsies) have been conducted according to the principles expressed in the Helsinki Declaration. All patients provided written informed consent as approved by the Helsinki Committee of the Israeli Ministry of Health and by the Kaplan Hospital H.C.

All animal work has been conducted according to the Weizmann Institute of Science's Institutional Animal Care and Use Committee (IACUC) and International Guidelines.

### Cell Culture

Mouse muscle progenitor cells were prepared as previously described [16]. Cells were passaged every 3 days or kept frozen. Cells from the 10–15 passages were used in the present investigation. Several clones were obtained from ROSA26 mice, which contain a transgene encoding a ubiquitously expressed bacterial β-galactosidase [17]. Other clones were derived from the Thy1-YFP 2.2 mice, which express YFP under a neuronal specific promoter [18]. The mouse myosphere clones that were used in the present study for intra-brain injection reproducibly gave rise to cells that spread and transdifferentiated as described later. Preliminary screening revealed some myosphere derived clones that did not yield transdifferentiating cells following their inoculation into the newborn brains. These clones were not used for the present study.

Human muscle biopsies (two patients; each biopsy less than one cm^3^) were taken by an orthopedic surgeon during surgery of bone or muscle, performed due to reasons unrelated to the biopsies for the current research.

The biopsies were dissociated as described for mouse muscles [16]. Several fractions of cells were collected by differential plating [8] on the basis of their adhesion properties. The fraction of cells that attached to the plates between 2 to 18 hrs after plating consisted of almost pure population of myogenic cells and was used for the preparation of primary human myoblasts cultures. For cloning, cells were diluted and plated at ∼2 cells/cm^2^. Colonies originating from single cells were collected, and propagated on gelatin coated plates. The work with human muscles was approved by Helsinki Committee of the Israeli Ministry of Health.

### Cells Transplantation into the Brain of Mice

Cloned cells were collected by trypsinization, washed with phosphate-buffered saline (PBS), and re-suspended at a concentration of 10^5^ cells/1.5 µl in cold PBS. Three day old C57BL mice were anesthetized, and 10^5^ cells were injected into their brain lateral ventricles, using a Hamilton syringe. Human cells were labeled with Hoechst prior to injection; the cells were suspended for 10 min at 37°C in PBS containing 0.02 mM Hoechst, and washed 3 times with PBS. Mice were sacrificed at the indicated time points, and their brains were removed for further analysis. The brains were fixed with 2.5% Paraformaldehyde (PFA) for 2–3 hours, transferred to 15% sucrose solution with 1% PFA, and incubated at 4°C for not less than 16 hrs. Sagittal brain slices of 25–40 mm were collected using sliding microtome (Leica SM 2000r). Five to seven mice were injected in each experiment. At least 3 brains were thoroughly sectioned and analyzed. The brain inoculations of β-gal marked mouse myosphere cells experiments were repeated at least 3 times and the injection of human myoblasts experiments were repeated twice.

#### X-Gal Staining

Slices were washed with PBS and stained overnight at 37°c with X-gal solution, as previously described [19]. Slices were washed with PBS, and counterstained with nuclear fast red.

### Immunochemistry

#### Cell cultures

Adherent cells were grown on gelatin or fibronectin coated glass cover slips. The immunostaining was done as previously described [16]. Pictures were taken with a 1310 digital camera (DVC).

#### Brain slices

Sections adjacent to X-gal positive slices were selected for immunohistochemical analysis. Slices were blocked and stained using M.O.M kit solutions (Vector, U.K). The slices were incubated over-night at room temperature with anti-b-gal (1∶200, Sigma-Aldrich, IL) together with one of the following antibodies: anti Doublecortin (1∶100, Santa-Cruz Biotechnology, Inc., CA, U.S.A), Tuj1 (1∶400, Covance, CA, U.S.A), NF-160 (1∶800, Abcam, U.K), NeuN (1∶200, Chemicon, CA, U.S.A). Secondary antibodies were either used for direct detection (Cy3 anti-mouse for the β-gal labeling), or with biotin-streptavidin-FITC for the neuronal markers. Slices were stained with DAPI solution for 2 min, air-dried and mounted as above. For the detection of NeuN, X-gal stained slices were labeled with anti-NeuN and detected with Vectastain ABC Kit (Vector, U.K). Brains injected with human cells were sliced, and slices containing Hoechst labeled cells were immunostained with the human specific anti-NF 70 Ab (1∶100, Chemicon, CA, U.S.A).

### FACS Analysis

For each experiment, one litter of 6–7 newborn mice was used. Brains were removed one week after injection of donor cells, and suspended according to Roy *et al.,* [20]. As most of the injected cells did not reach the cerebellum it was removed, and the remaining brain was minced with a scalpel, and then transferred into a solution containing 11.5 U/ml papain (preinduced with cysteine), and DNase (10 u/ml). The samples were rotated for 30 min at 37°C. Brains of 6–7 non-injected newborn mice served as a control. The cells were collected, and re-suspended in PBS. Hoechst was added (0.02 mM), and the cells were incubated for 10 min at 37°C, washed twice with PBS, and re-suspended at a concentration of 10^7^ cells/0.5 ml.

10^4^ cells of the main population were collected from each sample, to determine the cell-cycle of host neuronal cells. The rest of the population was sorted, and only the gated, YFP positive cells were collected, and cell-cycle analyzed. The experiments were done using either FACSVantage or LSR FACS (BD).

## Results

### Muscle Progenitor Cells Express Neuronal Markers Following Inoculation into the Brain of Newborn Mice

We have previously described the isolation and propagation of a population of myogenic cells, from adult mouse skeletal muscle, which proliferate as suspended clusters of cells (myospheres). These cells express the myogenic markers MyoD and desmin, indicating their commitment to the myogenic lineage. Under appropriate culture conditions, the myspheres adhere to the plate, start to spread out and form very thin mygenin and myosin heavy chain positive contractile fibers (needles). These needles are initially mononucleated but later fuse and form multinucleated fibers. When injected into injured muscle, the cells participated in muscle regeneration and gave rise to mature multinucleated muscle fibers [16]. We have isolated several clones of such muscle progenitor cells (MPCs). All of them express myogenic markers and differentiate to muscle fibers *in vitro*. Repeated reclonization analysis and staining for MyoD and desmin confirmed the stable retention of the myogenic commitment by virtually all cells. PCR analysis did not detect expression of Oct4, a hallmark of pluripotent embryonic stem cells.

**Figure 1:**
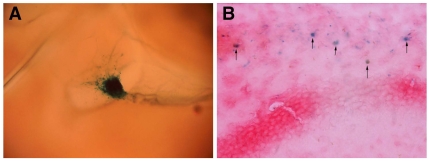
X-Gal staining of brains injected with cloned myosphere cells. Cloned MyoD positive myosphere cells (MPCs), obtained from ROSA26 mice, were injected into the lateral ventricles of the brain of newborn mice. Mice were sacrificed after 2 days (A), and 7 d (B) and their brains were removed. X-Gal staining was performed on the whole brain (in A), and on brain slices (in B). Two days after injection, the cells were still localized in the ventricle, in a compact cluster, while few cells started to migrate out of the ventricle (A). After seven days, the cells were detected as single blue cells in several brain regions, including the corpus callosum (B) Magnifications: A, ×40; B, ×200.

**Figure 2:**
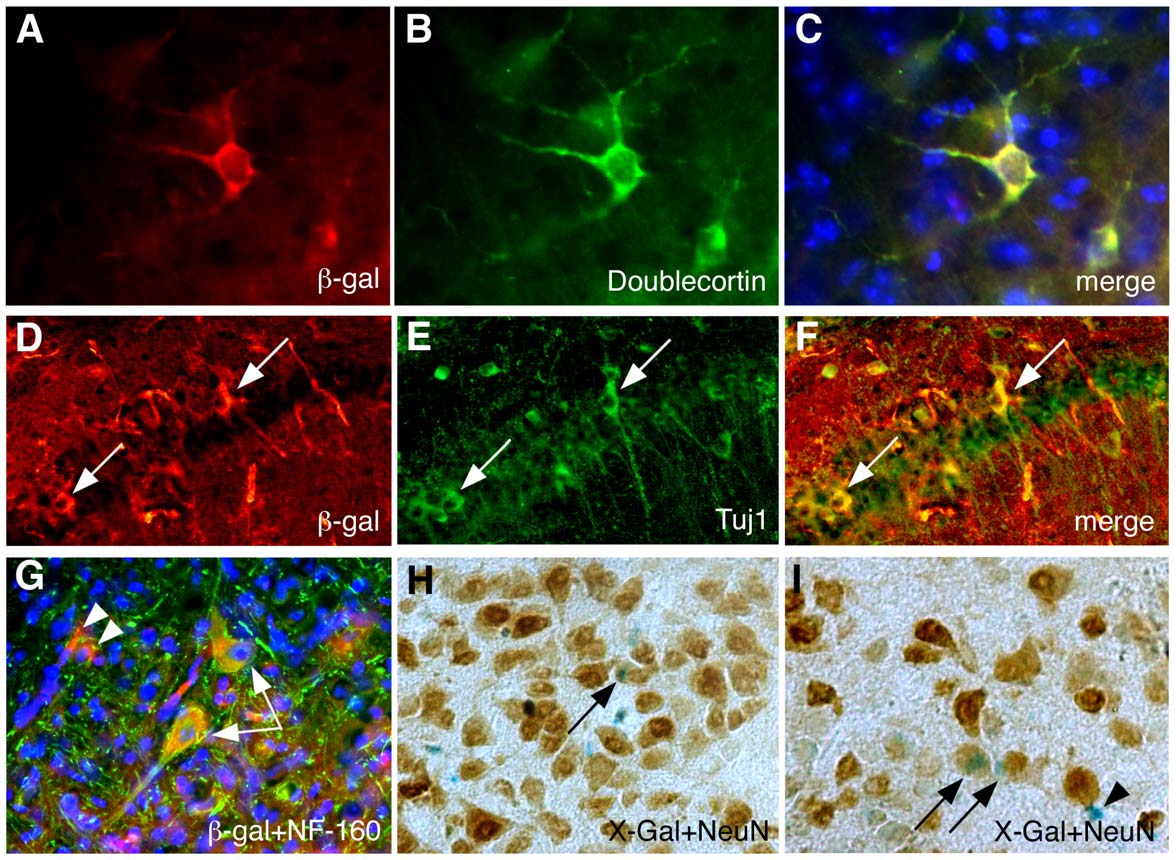
MPCs express neurogenic markers following inoculation into the brain of newborn mice. Brains were injected as described in Figure 1. A–F, seven days later the brains were fixed and sliced. A–C, a cell expressing both the donor marker, β-gal (red) and the early neuronal marker Doublecortin (green). D–F, cells expressing both β-gal (red) and early neuronal marker b-III-tubulin (Tuj1) (green). Arrows indicate double-labeled cells in the CA1 region of the hippocampus. G–I, Brains collected 2 weeks after injection. G, Double-immunostaining with anti-β-gal (red) and anti-mature neuronal marker NF-160 (green) revealed yellow stained cells that expressed both markers (arrows). Arrowheads in G indicate cells that express β-gal and not NF-160. H–I, X-Gal stained slices (blue) incubated with anti-NeuN antibody (mature neuronal marker). Labeling was detected using peroxidase staining (brown). Arrows indicate cells that express both NeuN and β-gal. Magnifications: A–C, G ×400; D–F, H–I, ×200. Arrowhead indicates a cell that expresses β-gal but not NeuN.

**Figure 3:**
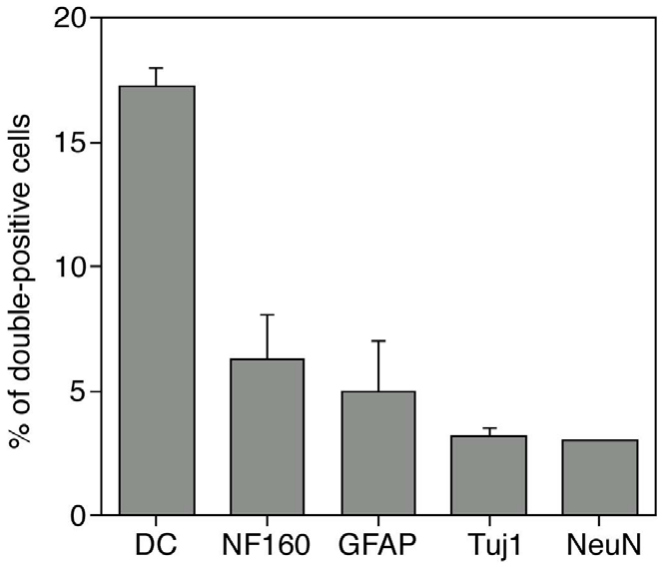
Quantification of brain inoculated MPCs expressing both donor β-gal and CNS cells marker. Double immunolabled cells were counted for each of the indicated antibodies. The bars describe the % of double-labeled cells, out of the detected, donor derived β-gal expressing cells that spread in the brain.

Since the brain of newborn mice continues to develop during the first two weeks post partum, we injected the cloned myogenic cell populations into the lateral ventricles of the brain of three day old mice, and followed their fate. The cloned cells were obtained from ROSA26 mice, which ubiquitously express β-gal [16], [17], therefore we first identified the location of the injected cells by X-gal staining (Fig. 1). One week after the injection, we observed extensive dispersion of the cells in the brain. Stained cells were mainly found in the cortex, corpus-callosum, hippocampus, and few cells were also observed in the thalamus, cerebellum, and the olfactory bulb. Immunofluorescence labeling with both anti-β-gal and anti-doublecortin (a marker for immature neurons) revealed a fraction of cells with a neuronal morphology that expressed both markers (Fig. 2A–C). Staining for another marker for immature neurons, b-tubulin-III (Tuj1), together with anti-b-gal, revealed double stained cells in the corpus callosum and in the CA1 field of the hippocampus (Fig. 2D–F). Staining for markers of mature neurons, NF-160 and NeuN also revealed a fraction of cells that expressed β-gal and the neurogenic marker (Fig. 2G–I). Overall, between 2000–4000 donor cells (expressing β-gal) were found in slices of each of the injected brains. About 17% of them expressed doublecortin, 6% expressed Neurofilament (NF)-160, 3expressed β-tubulin-III, and 3% expressed NeuN. About 5% of donor cells expressed the glial fibrillary acidic protein (GFAP) (Figure 3).

Analysis of MyoD expression by the injected cells revealed that only the cells in the vicinity of the injection site retained its expression, whereas the cells that spread in the brain and expressed neuronal markers did not express MyoD (not shown).

### Injected Myogenic Cells Express YFP Controlled by a Neuronal Specific Promoter

We have also cloned myosphere cells from skeletal muscles of transgenic mice harboring a yellow fluorescent protein (YFP) transgene under the control of regulatory elements of the Thy1 gene (Thy1-YFPH 2Jrs/J) that confer specificity of YFP expression to a subset of neurons [18]. The cloned cells (which, like all muscle cells of the transgenic mice, did not express YFP during proliferation and differentiation in cell cultures), were injected into the brain lateral ventricles of newborn C57BL mice. After one week, the injected brains were sliced and examined (without immunostaining) for the expression of YFP. We found that the pattern of distribution of injected cells that expressed YFP was very similar to that of myosphere cells co-expressing the neuronal markers and β-gal, described above (Fig. S1). This further supports the conclusion that myogenic progenitor cells are induced to express neuronal genes in the developing mouse brain.

### Human Myogenic Cells Spread in the Mouse Brain and Express a Human Neuronal Marker

**Figure 4:**
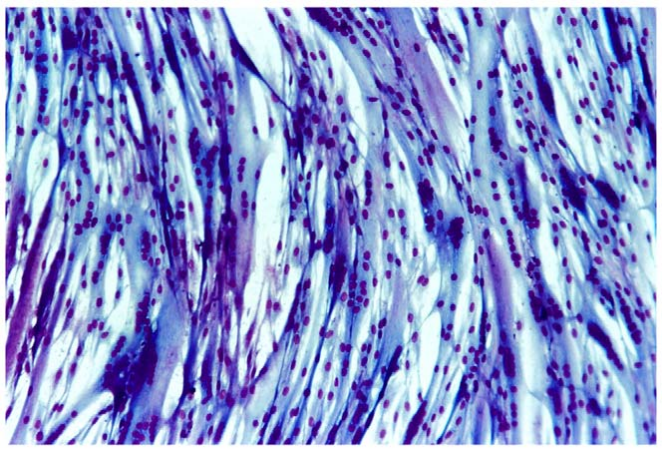
Cloned human myogenic cells. The culture was prepared as described in Method, grown for 10 days in the growth medium (BioAmf-2) and then induced to fuse and differentiate by changing to DMEM containing 10% horse serum, and insulin (4 units/100 ml). After 7 days the cells were fixed with methanol and stained with Giemsa. Note massive cell fusion. (Cells for inoculation into the developing mouse brains were collected before the change to the fusion inducing medium).

Using an adaptation of a differential pre-plating technique [8] we obtained from human muscle, cultures consisting of almost pure populations of MyoD expressing myogenic cells. These cells proliferate as a monolayer of myoblasts, which later fused into a network of multinucleated fibers. A myogenic clone isolated from such a culture (Figure 4) was used to test the capacity of human derived mononucleated myoblasts to give rise to neurogenic cells following injection to the brain of three day old mice. The cells were labeled with Hoechst dye prior to injection, and as described for mouse myosphere cells experiments, the inoculated brains were fixed and sectioned 5 and 9 days following injections. Sections showing Hoechst labeling were stained with an antibody specific for the human NF-70. The injected cells spread in the brain and were mostly localized close to the ventricles, in the subventricular zone (SVZ), and along the corpus callosum, in a pattern very similar to the lateral cortical stream (LCS) of migration of innate neurons, that occurs during embryogenesis [21]. The number of brain cells expressing human NF-70 was greater in the brains collected 9 days following injection (Figure S2) than in brains collected 5 days following injection.

These results show that like mouse myoblasts, also human myogenic cells migrate in the developing mouse brain and express a human neuronal marker, pointing at the generality of the phenomenon and its possible potential clinical application. Furthermore, since the human cell preparations consisted of cloned populations derived from conventional primary myoblast cultures, these data demonstrate that the conversion of myogenic cells to neuronal cells is not restricted to cells derived from myospheres.

### Transdifferentiation of Myogenic Cells to Neurogenic Cells Does Not Depend on Fusion with Host Cells

Several earlier studies, demonstrating the incorporation of donor cells in host tissues and transdifferentiation, were explained by fusion of donor and host cells and the formation of heterokaryons [e.g. 5], [6]. In contrast, in our study, most injected myogenic cells expressing neuronal markers contained only one normal looking nucleus. Only about 2% of the double-labeled cells had two nuclei or an aberrant nucleus. This suggested that the expression of the neurogenic markers in donor-derived cells was not the result of fusion with host cells.

**Figure 5:**
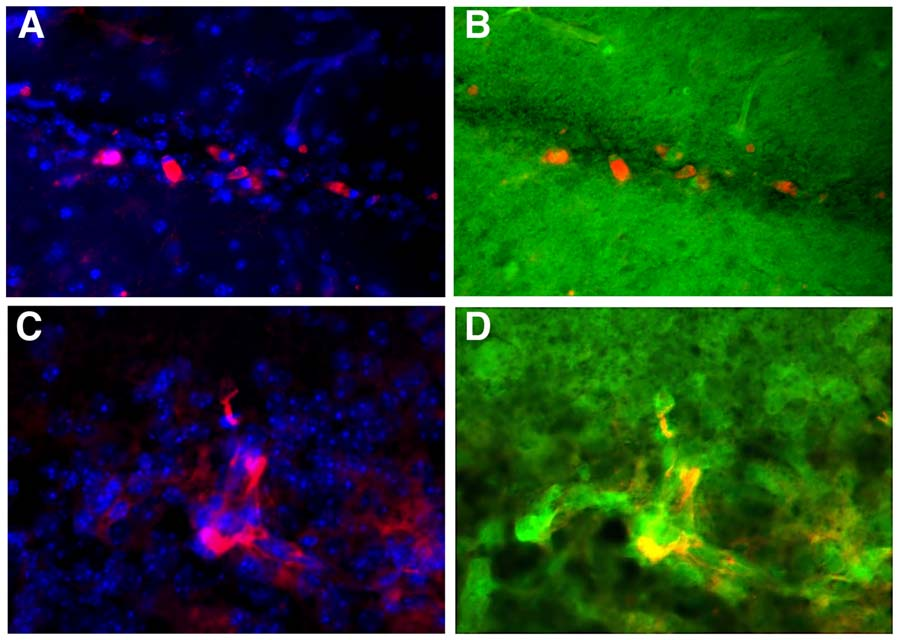
Donor injected myogenic cells do not fuse with host cells. Human myogenic progenitor cells, labeled with Hoechst, were injected into brains of mice ubiquitously expressing GFP. After 9 days, the brains were sliced and immunostained with a human specific anti-NF-70 antibody (red). Donor cells expressing human specific NF-70 protein (A). Merge images showed that these cells do not express the host GFP protein (B). C, D, Rare fusion events, usually resulted in the formation of a small cluster of bright yellow aberrant figures in the merged picture (D). Magnifications: ×200.

Consistent with this, when we injected human myoblasts into the brain of three day old mice ubiquitously expressing GFP, almost all donor cells that expressed human NF-70 did not express GFP (Fig. 5).

**Figure 6:**
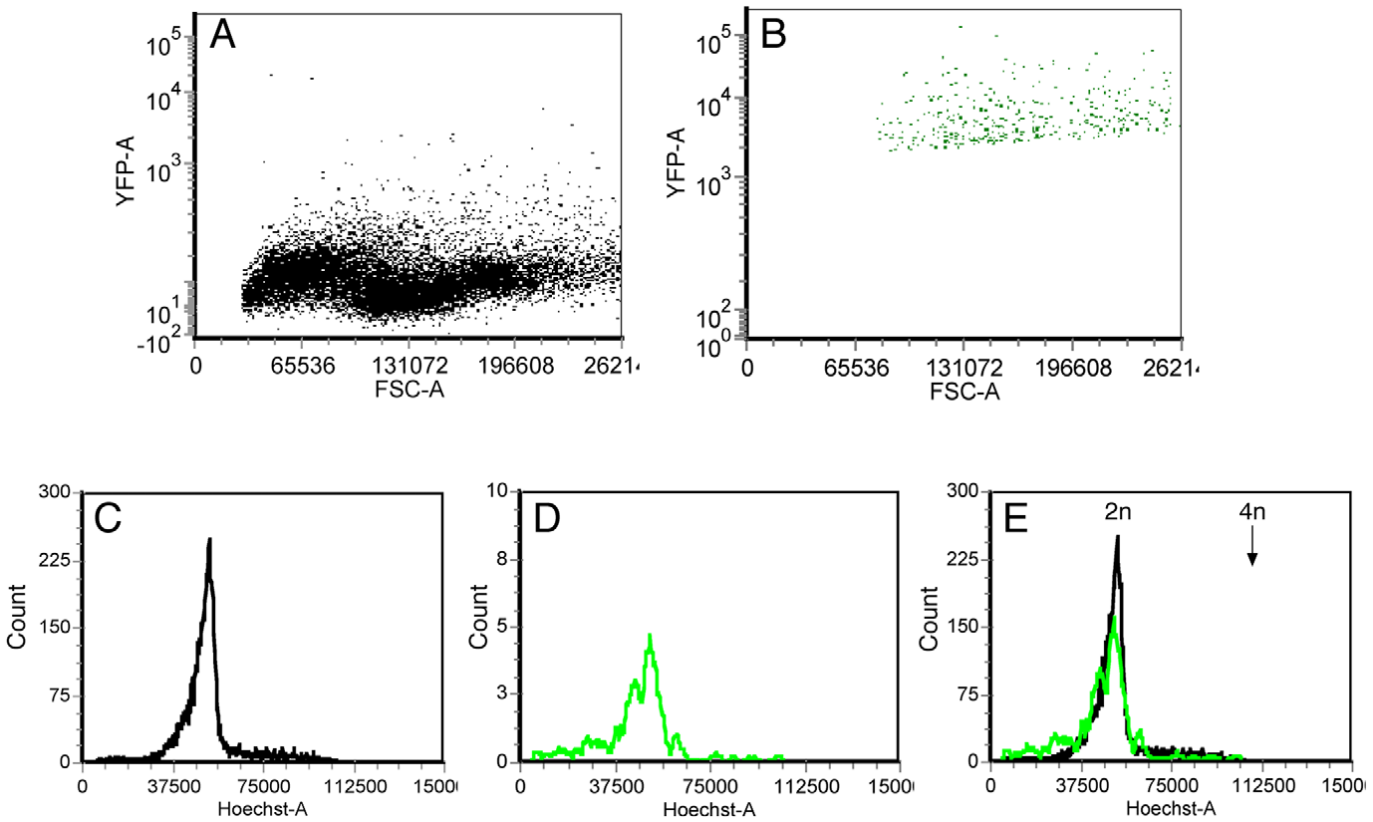
The DNA content of donor-YFP expressing cells is very similar to the DNA content of host diploid brain cells. Brains of wild type C57BL 3 day old mice were injected with myogenic clones derived from transgenic mice expressing YFP under the control of a neuronal specific promoter (Fig. S1). One week following injection the brain cells were suspended, labelled with Hoechst, and sorted by FACS. (**A**) The main population, containing brain cells that do not express YFP. (**B**) Cells expressing YFP. (**C–E**), Cell cycle analysis of the cells collected in **A** and **B**. The peak of the G1-G0 cells of the main population (C) and the pack of the G1-G0 cells of the YFP expressing cells (**D**) have the same value of Hoechst staining. The histogram of the YFP expressing cells was normalized to that of the main population, and overlapped, to demonstrate the very similar value of the G1-G0 populations (**E**). The 4n arrow indicates the calculated DNA value for tetraploid nuclei.

To investigate the question of cell fusion more rigorously, we inoculated the cloned myosphere cells derived from the Thy1-YFP transgenic mice (described above) into the brains of 3 day old wt mice. One week later, the brains were dissociated, and the cells were labeled with Hoechst, to measure their DNA content. Approximately 500 YFP expressing cells were sorted by FACS, and analyzed for their DNA content. Cell cycle analysis of these cells revealed that the DNA content of the donor derived YFP expressing cell population was very similar to that of the main population (Fig. 6). There was no evidence for a peak that indicates tetraploidy. This result indicates that the expression of neuronal traits by the cells of donor origin was mainly not due to fusion with host cells, thereby implying a genuine transdifferentiation of the injected cells, which was induced by the host environment.

## Discussion

Experiments with cloned mammalian cells demonstrated the very stable retention of differentiation programs during extended periods of cell proliferation in culture. In contrast, cell fusion, nuclear transplantation, and forced expression of transcription factors, demonstrated the plasticity of nuclei of differentiated cells [1], [22]–[25]. However, the question is still open of whether intact genetically unmodified somatic cells, committed to specific differentiation fates, can be reprogrammed *in vivo* by host ectopic micro-environment [e.g. 2]–[6], [26], [27]. The major controversial questions are a) the possible heterogeneity of the donor cell population and b) whether it is a genuine induction process that occurs in response to the host environment, or a result of fusion between host and donor cells. We addressed these questions using cloned myogenic progenitor cells, committed to the myogenic lineage. Injection of such cells into the brain of mice ubiquitously expressing GFP enabled us to determine whether cells that express both the donor marker and neuronal markers, also express the host specific GFP protein. Using another approach we measured the DNA content of sorted donor cells expressing YFP controlled by a neuronal specific promoter, and compared their DNA content with that of the main brain population. Both techniques indicated that most transdifferentiated cells did not fuse with host cells.

Repeated experiments revealed a reproducible time-course of the process. Two days after the injection, the majority of the cells still remained in the ventricle. A massive migration out of the ventricle occurred at the fourth day (not shown), and peaked at the seventh day. The highest percentage of donor cells expressing neuronal markers was observed 7 days and 9 days post injection. Two weeks after injection, there was a decrease in the frequency of donor cells. Brains analyzed 30 days post injection revealed a further reduction in the frequency of donor cells (not shown). The reason for this apparent reduction is unknown. Although ROSA 26 mice are on a C57BL/6 background and the brain is a relatively immunological privileged site, an immunologic reaction cannot be excluded. This observation needs further investigation.

The rarity of the donor cells rendered it impossible to test their electrophysiological activity in vivo. However, immunocytochemical examination demonstrated the presence of donor-derived cells expressing markers of mature neurons, such as NF-160 and NeuN, and many of the cells gained a neuronal morphology. This suggests that at least a fraction of these cells reached neuronal maturity.

In view of recent progress in the refinements of culture conditions for controlling the differentiation of embryonic and adult stem cells *in vitro*, it is of obvious interest to search for conditions that recapitulate in cell culture the conversion of myogenic cells to neurogenic cells and to test the functionality of the transdifferentiated cells, as indicated by their capacity to form neuromuscular junctions and by recording their electrophysiological activity.

The present investigation suggests that the developing brain emits signals that stimulate cell migration and transdifferentaion of the donor derived cells. It is of interest to note that inoculation of myosphere cell populations into adult brain resulted in very little donor cell migration and undetectable transdifferentiation. However, myosphere cells inoculated into the damaged brain of Experimental Autoimmune Encephalitis (EAE) affected mice, the mouse model of multiple sclerosis, did spread in the brain and a fraction of them gained neuronal morphology and expressed neuronal markers [28]. This suggests that the regenerating brain (but not normal adult brain) emit a signal or signals with similar functions. Moreover, the present study indicates that human myoblasts respose to the mouse signal.

Sigurjonsson et al., [29] reported that engrafting of CD34+ adult human hematopoietic cells in close proximity to an injured spinal cord of a developing chick embryo, resulted in the efficient participation of the human derived cells in the regeneration of the chick embryo spinal cord, and their transdifferentation into functional neuronal cells. This suggests the wide-spread and strong evolutionary conservation of such signals.

In addition to the basic biological aspects, the migration of inoculated human myoblasts in the brain, and the expression of neuronal markers by a fraction of them, suggest a possible therapeutic potential, leading to the use of innate genetically unmodified myoblasts for autologous cell therapy of diseases of the CNS. The possibility to reprogram somatic cells into iPS cells, closely resembling embryonic stem cells, raised great hopes for a customized cell therapy approach, using autologous somatic cells converted into stem cells. Recent studies indicate that induction of pluripotency can also be done by exposing intact genetically unchanged somatic cells to chimeric transcription factors that penetrate the cells and induce an epigenetic reprogramming process leading to pluripotency [30], [31]. However, one of the main problems which may hamper these approaches is the tumorigenic potential of pluripotent cells [e.g. 32]–[35]. The present study supports the claim that committed somatic cells can be induced to transdifferentiate *in vivo* into a different cell lineage phenotype by ectopic environmental signals, thus, circumventing the use of embryonic stem cell like cells for autologous cell therapy. The short time it takes for the implanted donor cells to express neuronal markers, suggests a direct conversion of the myogenic cells into neuronal phenotype without the involvement of an embryonic stem cell state.

### Supporting Discussion

Steffel et al., [36] reported that cells of the myogenic line C2, injected into the brain of rat embryos contributed to mesodermal derived tissues such as endothelium, but no contribution to the neurogenic lineage was observed. In addition to the neuronal differentiation, described above, we also observed the incorporation of a fraction of injected cells into the brain vasculature (not shown). The difference between those results and the present study might be due to the difference in the donor cells (C2 vs myospheres cells) or in the nature of the host environment (i.e. embryonic rat brain vs newborn mouse brain).

## Supporting Information

Figure S1 Activation of a transgenic neuronal specific promoter in transgenic donor MPCs injected into the brain of wild-type, new-born mice. Cloned myosphere cells obtained from the Thy1-YFP transgenic mice were injected into the brains of 3 day old C57BL mice. The brains were removed one week following injection and sliced; selected slices were screened for the expression of YFP using fluorescence microscope. Part of the injected cells expressed YFP, thereby indicating that the neuron specific promoter was activated in those donor cells. The pattern of the distribution of these cells was very similar to the pattern observed with either X-Gal or β-gal immunoflourescence stainings. Nuclei were stained with DAPI. A,B, corpus-callosum, C,D, hippocampus. Magnifications: A-C, ×200; D, ×400.
(6.92 MB TIF)

Figure S2 Human myogenic cells migrate in the brain of newborn mice and express a human specific neuronal marker. Cloned human myogenic cells were labeled with Hoechst dye (blue) and injected into the lateral ventricles of newborn mice. Brains were removed after 9 days, fixed and sliced. The injected cells were localized mostly in the cortex, subventricular zone, and corpus callosum (A). Selected slices were immunostained with antibody specific to human NF-70. B-C donor injected cells expressing human specific NF-70 (green). D-E- higher magnifications of B-C. IS- injection site, SVZ-subventricular zone, CC-corpus callosum.
(3.88 MB TIF)
