# Supplementary figures and images for: Cloned Myogenic Cells Can Transdifferentiate In Vivo into Neuron-Like Cells
Source: PLoS One. 2010 Jan 21;5(1):e8814. doi: 10.1371/journal.pone.0008814 (PMC2809103; doi:10.1371/journal.pone.0008814)

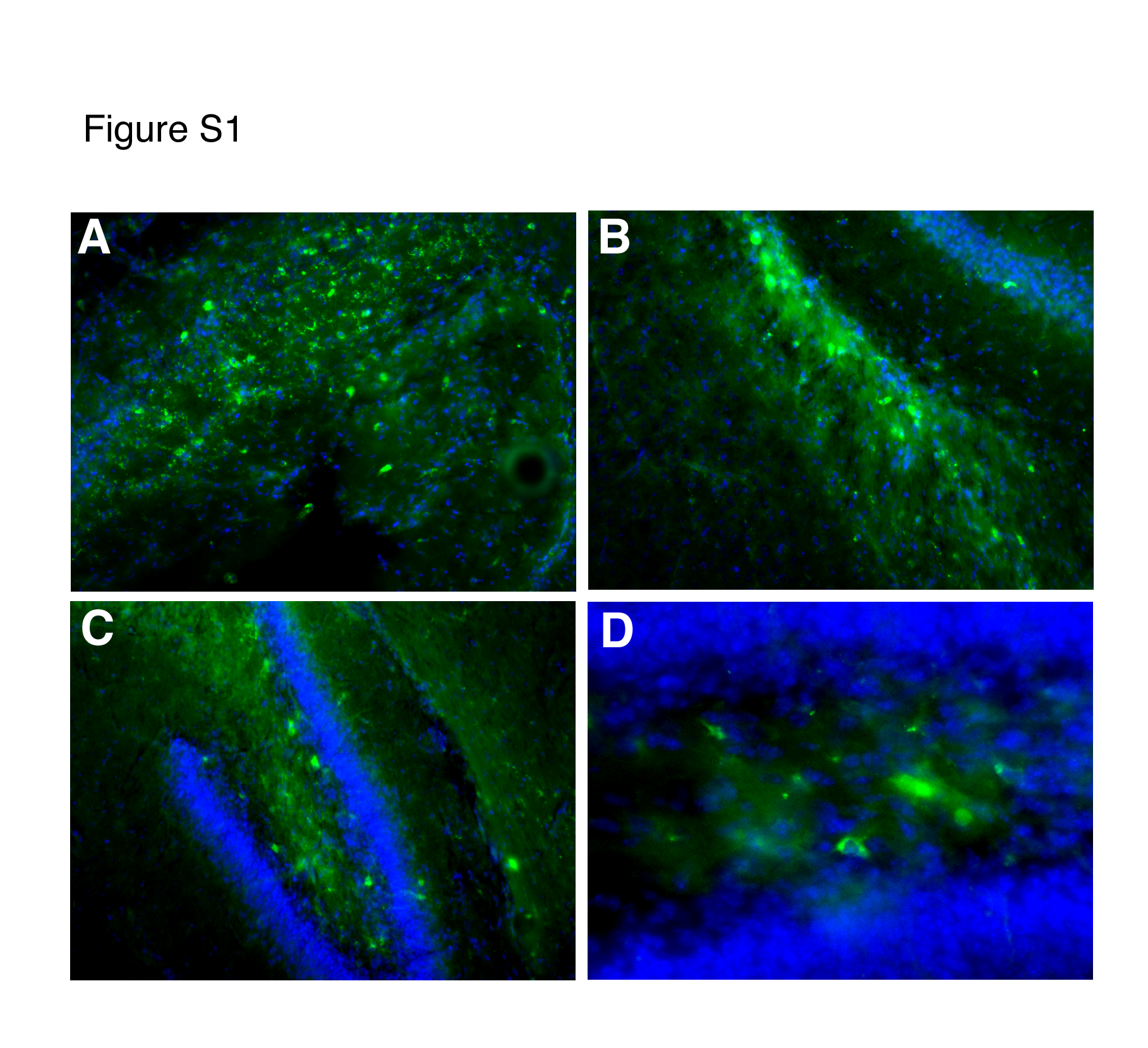

Supplement: Figure S1 — Activation of a transgenic neuronal specific promoter in transgenic donor MPCs injected into the brain of wild-type, new-born mice. Cloned myosphere cells obtained from the Thy1-YFP transgenic mice were injected into the brains of 3 day old C57BL mice. The brains were removed one week following injection and sliced; selected slices were screened for the expression of YFP using fluorescence microscope. Part of the injected cells expressed YFP, thereby indicating that the neuron specific promoter was activated in those donor cells. The pattern of the distribution of these cells was very similar to the pattern observed with either X-Gal or β-gal immunoflourescence stainings. Nuclei were stained with DAPI. A,B, corpus-callosum, C,D, hippocampus. Magnifications: A-C, ×200; D, ×400. (6.92 MB TIF) [file pone.0008814.s001.tif]

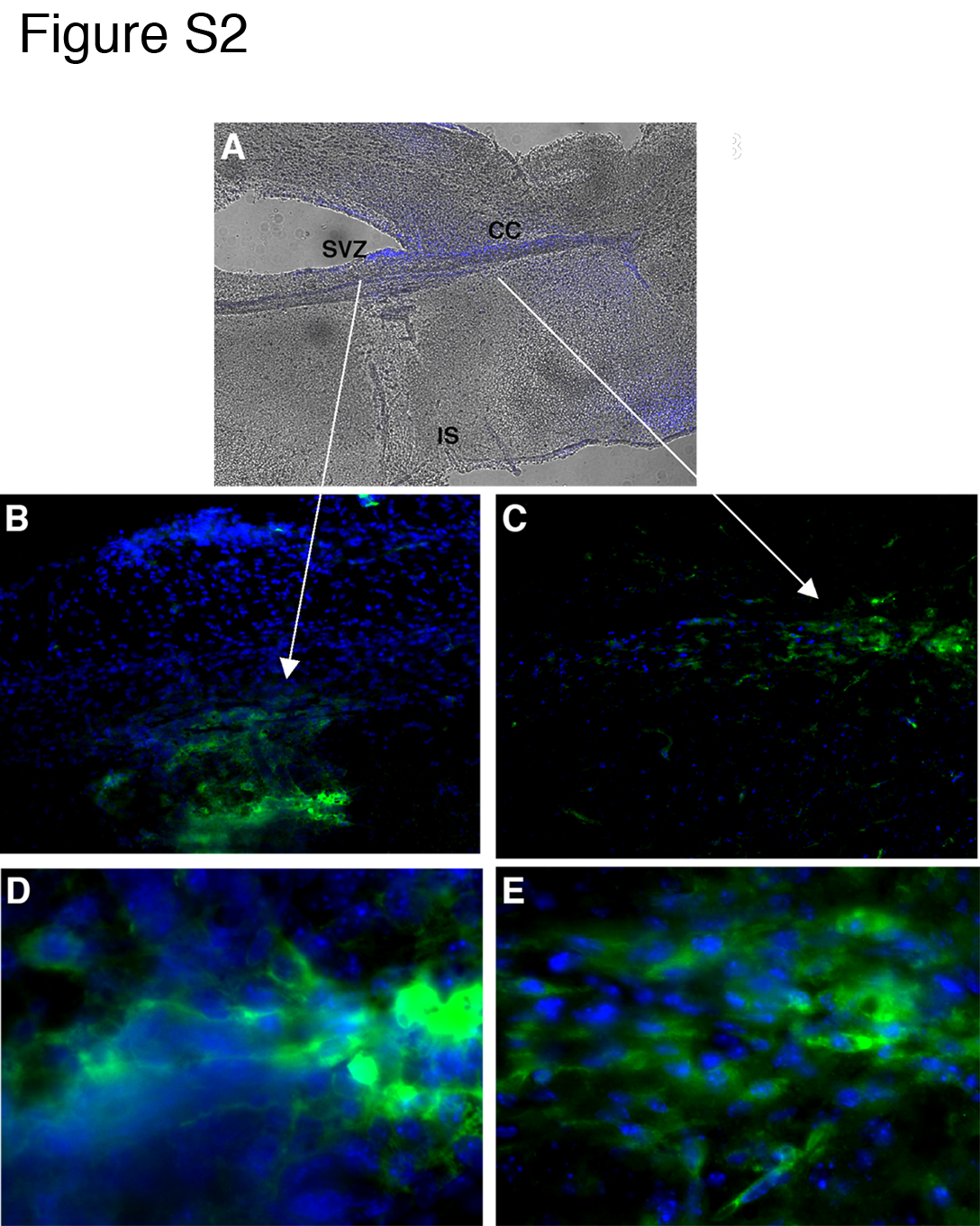

Supplement: Figure S2 — Human myogenic cells migrate in the brain of newborn mice and express a human specific neuronal marker. Cloned human myogenic cells were labeled with Hoechst dye (blue) and injected into the lateral ventricles of newborn mice. Brains were removed after 9 days, fixed and sliced. The injected cells were localized mostly in the cortex, subventricular zone, and corpus callosum (A). Selected slices were immunostained with antibody specific to human NF-70. B-C donor injected cells expressing human specific NF-70 (green). D-E- higher magnifications of B-C. IS- injection site, SVZ-subventricular zone, CC-corpus callosum. (3.88 MB TIF) [file pone.0008814.s002.tif]
